# Valproic Acid-Induced Myoclonus in a Demented Patient: A Case Report

**DOI:** 10.1155/2009/392091

**Published:** 2009-08-09

**Authors:** Tina M. Gardner, Rehan Aziz, Sunanda Muralee, Rajesh R. Tampi

**Affiliations:** Department of Psychiatry, Yale University School of Medicine, New Haven, CT 06510, USA

## Abstract

Valproic acid and its derivatives are now commonly used to treat various psychiatric disorders in the elderly. Data indicates that the elderly patients are more susceptible to developing neuropsychiatric complications when treated with these medications. In this report, we describe the case of a 66-year-old woman with early-onset, Alzheimer's type dementia, who developed myoclonus when treated with a valproic acid preparation for behavioral disturbances associated with the dementia.

## 1. Introduction

Developed originally as an anticonvulsant medication, the use of valproic acid (VPA) in patients with psychiatric disorders has increased in recent years [1]. Given the current concerns regarding the use of the antipsychotic medications in older patients with dementia, there is interest in using mood stabilizing anticonvulsants for demented patients with agitation and behavioral problems [2, 3]. We are reporting the case of a woman with early-onset, Alzheimer's type dementia, who developed an adverse reaction to a valproic acid preparation when used for treating behavioral problems associated with the dementia. Although myoclonus in the absence of ammonemia and normal valproic acid level has been reported in a 42-year-old depressed patient treated with VPA [4], from our review of literature, this is the first reported case of myoclonus due to valproic acid in a patient with dementia when used to treat behavioral disturbances.

## 2. Case Report

Ms. JB, a 66-year-old Caucasian woman, was admitted to our inpatient geriatric psychiatry unit from her long term care (LTC) facility because of behavioral disturbances associated with her dementing illness. She was resistant to care, was having persistent vocalization, and was hallucinating. Functionally, she required full assistance with all her ADLs. Her past medical history included early-onset dementia of Alzheimer's type, severe, hyperlipidemia, hypertension, and dysphagia. The patient did not have a history of psychiatric illness including anxiety, psychosis, substance abuse, or psychiatric hospitalizations.

Her medications at admission to our unit were multivitamins, citalopram 20 mg po daily, simvastatin 40 mg po daily, Vitamin D3 1000 IU po daily, calcium carbonate 600 mg po bid, valproic acid sprinkles 375 mg po twice daily, docusate 100 mg po twice daily, and risperidone 0.5 mg po twice daily.

Our review of record, indicates that the patient was being treated with citalopram, valproic acid sprinkles, and risperidone for the behavioral disturbances associated with her dementia. It is unclear exactly as to when the valproic acid sprinkles was started, but it appears that the patient was taking this drug for at least the last month prior to her hospitalization.

On admission examination, the patient was noted to be perseveratively calling out and crying. Her vital signs were stable, and she was uncooperative to the examination. She was underweight, at 66 inches in height, 99 lbs in weight, and body mass index (BMI) = 16 kg/m^2^. Despite her history of hypertension, her blood pressure (BP) on admission was low at 96/57 mm hg. She was noted to have a dysconjugate gaze. She had marked jerking movements of her upper extremities consistent with myoclonus. Muscle tone was increased, and it was difficult to assess for cogwheeling, as patient was unable to voluntarily relax her extremities. Myoclonus in her lower extremities was also noted when her feet were dorsiflexed. She was disoriented to time, place, and person and was unable to participate in a formal cognitive testing.

Laboratory examination on admission revealed a low serum albumin of 3.4 gm/dL. Her ammonia level was low normal at 4 umol/L. Her valproic acid level was 39 mg/L, a level generally considered to be subtherapeutic for patients with seizure disorders. Sodium was 143 meq/L, potassium was 3.7 meq/L, chloride was 104 meq/L, bicarbonate was 33 meq/L, blood urea nitrogen (BUN) was 12 mg/dL, serum creatinine was 0.7 mg/dL, glucose was 91 mg/dL, serum calcium was 8.5 mg/dL, aspartate transamisase (AST) was 7 iu/L, and alanine transaminase (ALT) was 21 iu/L. Vitamin B12, folate, and thyroid stimulating hormone (TSH) levels were normal. Urinalysis was unremarkable. Complete blood count (CBC) was normal with a white cell count of 8100/cmm, a hemoglobin level of 13.7 gm/dL, and a hematocrit of 41.2%.

Following the initial evaluation, the VPA dose was decreased and then discontinued, with complete resolution of her myoclonus. Quetiapine was started and titrated to 25 mg po at noon and 12.5 mg po at 5 pm for agitation. Because of her late afternoon agitation, risperidone 0.5 mg was increased from twice a day to 0.5 mg po three times a day, with the additional dose given in the early afternoon. Her restlessness, agitation, vocalizations, hallucinations and resistance to care were significantly decreased from admission on this medication regimen. She was discharged back to her long term care facility in a stable state.

## 3. Discussion

VPA is itself a simple fatty acid and can disrupt the urea cycle by causing impairment in the body's usual fatty acid metabolism [5]. The disruption in the urea cycle causes high serum ammonia levels [5]. Encephalopathy secondary to hyperammonemia caused by VPA has been reported by others [6, 7] as well as encephalopathy and myoclonus associated with VPA in the absence of a significantly elevated ammonia level [8]. Our patient's ammonia level was low normal, and her VPA level was not elevated. Her serum liver enzyme levels were normal. However, discontinuation of VPA was associated with a complete resolution of the myoclonus. Therefore, a serum level of VPA which would not normally be considered to be high appears to have been responsible for her myoclonus. Because valproic acid is a mitochondrial toxin, it can cause myoclonus even when it does not cause high serum ammonia levels [5, 9].

We considered delirium as a differential diagnosis for the patients behavioral disturbances that give her age and her history of dementia [10]. To this end we also completed a work up which was essentially normal. Although delirium remained a possibility, the lack of fluctuating levels of consciousness and no evidence for metabolic or infectious causes for her behavioral issues made this diagnosis less likely.

The patient came to us with a history of hypertension, but she was found to have several recordings of low blood pressures during her stay at our unit. After a careful review, we concluded that these blood pressure changes were due to autonomic failure associated with her severe dementia and the psychotropic medications that she was taking [11, 12]. Although she had her several blood pressure recordings that were low during her hospitalization, she also had several days where her blood pressure recordings were normal. Her blood pressures prior to discharge ranged 103–107/63–69 mm hg.

It remains unclear from literature as to when an elderly patient will be susceptible to the developing neuropsychiatric side-effects from VPA. However, available data indicates that all elderly patients treated with VPA should be closely monitored regardless of the duration of therapy and especially during periods of acute illness [13, 14]. For those patients who develop myoclonus despite close monitoring, the VPA should be discontinued immediately.

Elderly patients are very sensitive to side effects of medications [15]. In this patient, an anticonvulsant mood stabilizer, VPA, was given in an attempt to avoid the potential for the extrapyramidal and cerebrovascular side effects of antipsychotic drugs profiles. However, VPA was not effective in controlling her agitation and behavioral disturbances, and it caused its own untoward side effect of myoclonus. Although some psychotropic medications have been found to be helpful in the treatment of BPSD, none of them have proven efficacy and benign side-effect profiles [3, 16]. Elderly patients also have more medical comorbidities and are taking multiple medications [17]. This puts them at higher risk for developing medical complications and medication side-effects along with drug-drug interactions profiles [15]. These issues must be considered while giving a new medication to the older patient especially with dementia.
